# MarVis: a tool for clustering and visualization of metabolic biomarkers

**DOI:** 10.1186/1471-2105-10-92

**Published:** 2009-03-20

**Authors:** Alexander Kaever, Thomas Lingner, Kirstin Feussner, Cornelia Göbel, Ivo Feussner, Peter Meinicke

**Affiliations:** 1 Department of Bioinformatics, Institute of Microbiology and Genetics, Georg-August-University Göttingen, Göttingen, Germany; 2 Department of Developmental Biochemistry, Institute for Biochemistry and Molecular Cell Biology, Georg-August-University Göttingen, Göttingen, Germany; 3 Department for Plant Biochemistry, Albrecht-von-Haller-Institute for Plant Sciences, Georg-August-University Göttingen, Göttingen, Germany

## Abstract

**Background:**

A central goal of experimental studies in systems biology is to identify meaningful markers that are hidden within a diffuse background of data originating from large-scale analytical intensity measurements as obtained from metabolomic experiments. Intensity-based clustering is an unsupervised approach to the identification of metabolic markers based on the grouping of similar intensity profiles. A major problem of this basic approach is that in general there is no prior information about an adequate number of biologically relevant clusters.

**Results:**

We present the tool MarVis (Marker Visualization) for data mining on intensity-based profiles using one-dimensional self-organizing maps (1D-SOMs). MarVis can import and export customizable CSV (Comma Separated Values) files and provides aggregation and normalization routines for preprocessing of intensity profiles that contain repeated measurements for a number of different experimental conditions. Robust clustering is then achieved by training of an 1D-SOM model, which introduces a similarity-based ordering of the intensity profiles. The ordering allows a convenient visualization of the intensity variations within the data and facilitates an interactive aggregation of clusters into larger blocks. The intensity-based visualization is combined with the presentation of additional data attributes, which can further support the analysis of experimental data.

**Conclusion:**

MarVis is a user-friendly and interactive tool for exploration of complex pattern variation in a large set of experimental intensity profiles. The application of 1D-SOMs gives a convenient overview on relevant profiles and groups of profiles. The specialized visualization effectively supports researchers in analyzing a large number of putative clusters, even though the true number of biologically meaningful groups is unknown. Although MarVis has been developed for the analysis of metabolomic data, the tool may be applied to gene expression data as well.

## Background

Metabolomic profiling in general aims to identify or confirm biomarkers that are represented by specific metabolite intensity profiles in the context of different physiological and/or experimental conditions. These conditions may represent different phenotypes of a species, disease or environmental and genetic perturbations, or a time course comparing different developmental or physiological stages of an organism [1-4]. High-throughput analytical measurements, as obtained from mass spectrometry experiments [5,6], provide a large number of intensity profiles for accumulation of different metabolites. These data sets show an even higher complexity when repeated measurements for each condition have been performed. For an interpretation based on the experimental conditions these replicas need to be aggregated using e.g. the corresponding mean or median value. For comparative analysis of relative metabolite concentrations it is usually necessary to normalize the resulting intensity vectors, e.g according to a unit Euclidean or "city block" norm. In the following, the aggregated and normalized multivariate intensity profiles are referred to as marker candidates.

Clustering is a well-established technique in the context of gene expression analysis and coexpression studies [7,8]. Intensity-based clustering by analogy aims to group similar intensity profiles in order to identify interesting groups of marker candidates and visualize them in a convenient way. A major problem with the application of clustering algorithms is that an adequate number of clusters can often not be inferred automatically. A purely data-driven approach always bears the risk of over- or under-clustering because the correct number of clusters usually depends on task-specific constraints [9]. One-dimensional self-organizing maps [10] (1D-SOMs) realize a linear array of prototypes that correspond to local averages of the data, ordered according to their similarity. In metabolomic analysis the visualization of ordered prototypes provides a quick overview on relevant intensity patterns in the data and allows to easily merge neighboring groups of marker candidates into meaningful clusters. For example, in [11] we detected a significant number of clusters representing different physiological stages during a plant wounding time course as described in [12,13].

The 1D-SOM realizes a robust and reproducible ordering in particular with regard to changing data quality [11]. Unlike the classical two-dimensional self-organizing maps (2D-SOMs) [10], which are utilized in a number of software tools for gene expression analysis [14,15] and metabolomics [16,17], 1D-SOMs allow a simultaneous visualization of the clustering and the underlying intensity profiles by means of the topologically ordered prototype array. This visualization corresponds to a two-dimensional color-coded matrix, where the first dimension represents the prototype order and the second dimension represents the experimental conditions. While 2D-SOMs can be used to visualize the two-dimensional variation in a single condition, 1D-SOMs provide a complete view on the one-dimensional variation in all conditions simultaneously. Therefore, 1D-SOMs provide a convenient overview of highly complex metabolomic data sets. Beside a number of general software packages, like the well-known SOM toolbox [18] or the "Clustering for Business Analytics" and SOM packages for the R-project [19], several more specific tools [20-22] provide functions to order and visualize multivariate intensity profiles along a one-dimensional array. Though, none of them provides a specialized interface for convenient 1D-SOM visualization and analysis of metabolomics data.

In the following, we introduce the MarVis (**Mar**ker **Vis**ualization) tool, which implements the concept of 1D-SOM clustering and visualization. Based on an example workflow, the functionality and utility of MarVis is demonstrated.

## Implementation

MarVis was written in the Matlab^® ^programming language and has been compiled for Microsoft^® ^Windows XP/Vista and Linux x86. Execution of the software requires installation of the Matlab^® ^Compiler Runtime, which is provided with MarVis. The installation packages and the documentation can be downloaded from the project home page .

For data import and export MarVis uses the CSV (Comma Separated Values) file format, which can easily be processed by statistical analysis software and spreadsheet applications. Besides data set meta information and customizable headers, a CSV file for use with MarVis consists of marker candidate-specific lines. Each line contains data fields with intensity measurements for all conditions and replicas (for details see MarVis documentation). By default, MarVis performs an aggregation of repeated measurements for each condition using the corresponding mean or median value. The resulting intensity vectors are normalized before clustering using the Euclidean or "city block" norm or a z-score transformation. If alternatively normalized intensity profiles should be used for clustering, these user-normalized profiles can be stored as additional data in the CSV file. It is also possible to store additional marker candidate properties, which are displayed by MarVis as text. For high-contrast visualization of prototype and marker candidate profiles MarVis uses customizable colormaps, which map original and normalized intensity values to a broad color spectrum. The colormapping for original and normalized intensities is calculated independently according to the respective minimum and maximum intensity values.

## Results

In the following, the functionality of MarVis is demonstrated on the basis of a metabolomic case study of a plant wounding experiment analyzed by ultra performance liquid chromatography coupled with an orthogonal time-of-flight mass spectrometer as described before in [11]. The data set contains 837 marker (metabolite) candidates for the wound response of the thale cress *Arabidopsis thaliana *under 8 conditions. The first four conditions reflect the metabolic situation within a wounding time course of wild type (wt) plants starting with the control plants followed by the plants harvested 0.5, 2 and 5 hours post wounding. The conditions 5 to 8 represent the same time course for the jasmonate deficient mutant plant *dde *2-2 [23]. Each condition contains 9 replica samples. The corresponding data set is supplied with the MarVis tool as a CSV file (example data set 1).

### File import

The data set is imported using the **Open for clustering** entry in the File menu. After choosing the input file (examples/dataset1.csv), MarVis displays the **Import dialog**, where the delimiter character (comma), the start row (5) and column of the header (3), the number of conditions (8), and the number of samples for each condition (9) are specified. In this example, we use the mean intensity value for aggregation of replicas and the Euclidean norm for normalization of intensity profiles (checkbox **Import normalized markers** deactivated, radio buttons **mean** and **2-norm** selected).

### Clustering

**Figure 1:**
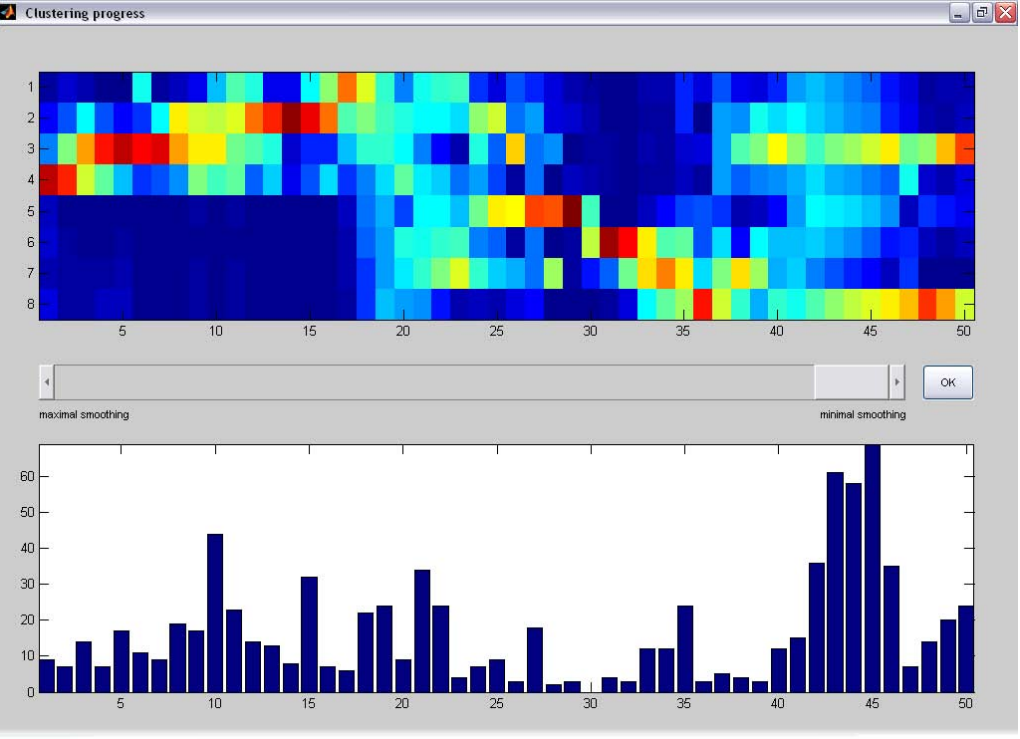
**Clustering progress window (final state)**. The clustering progress window after completion of the clustering process. The scrollbar in the middle of the window may be used to browse through intermediate clustering results with a higher smoothing over the prototype profiles. In the upper plot, MarVis displays the prototypes of the selected clustering state according to the current colormap. By default, MarVis uses the Jet colormap, i.e. red colors represent high intensities and blue colors represent low intensities. The vertical axis represents the different data set conditions, the horizontal axis corresponds to the prototype numbers. In the lower plot, the number of marker candidates that are associated with each prototype are represented as vertical bars with different height.

After confirmation of the import options, the **Clustering dialog** is opened. Here, a title (dataset1.csv) and the number of prototypes (e.g. 50) have to be specified. MarVis starts the iterative clustering process and displays the intermediate prototype intensity profiles for each clustering state and the number of currently associated marker candidates in a separate window. The clustering process for the example data set only takes a few seconds on a standard PC. After the clustering process has been finished, the clustering state can be selected according to the desired degree of prototype smoothing (see [11]) by adjusting a scrollbar (see figure 1). Here, we choose the final clustering state corresponding to a minimal amount of smoothing, which is suitable for analysis in most cases.

### Visualization and analysis

**Figure 2:**
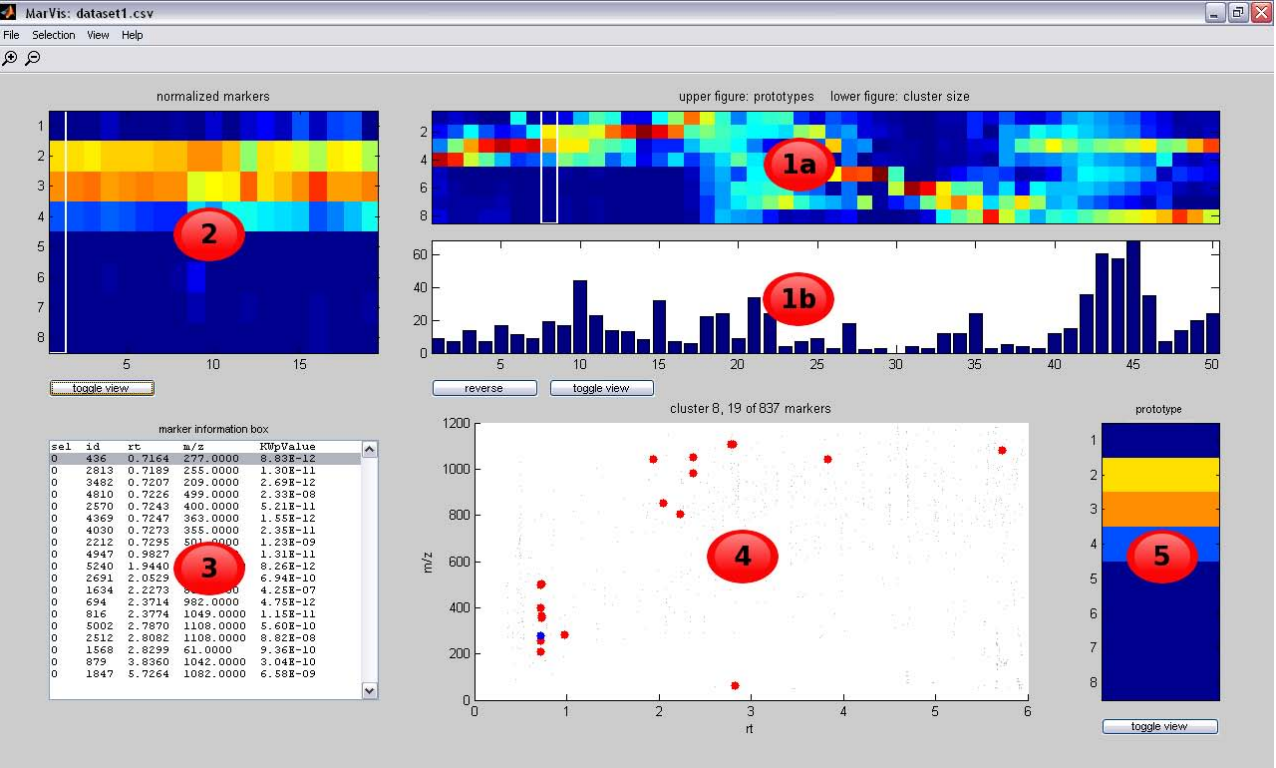
**MarVis main window**. The main window of MarVis after selecting a cluster/prototype for further analysis. The window is divided into several regions, which display different information: The prototype plot (compare with figure 1) shows the array of prototypes according to the current colormap (region 1a) and the number of marker candidates in the associated clusters (region 1b). The cluster plot (2) displays the intensity profiles of marker candidates in the currently activated cluster where a white rectangle marks the currently activated candidate and the associated prototype. The marker information box (3) shows detailed information of all candidates in the currently activated cluster. The marker scatter plot (4) displays the retention time vs. the mass-to-charge-ratio of each marker candidate in the currently activated cluster using big red dots. The currently activated candidate is represented by a big blue dot. In the background all marker candidates of the current data set are plotted as small gray dots. The active-prototype/marker plot (5) displays the magnified prototype profile of the activated cluster according to the current colormap.

After selection of an appropriate clustering state for analysis, MarVis returns to the main window and displays the prototype profiles and the number of associated marker candidates in the upper right region. After mouse click on a column corresponding to a particular prototype, further information regarding this prototype is displayed in the other regions of the main window (see figure 2).

**Figure 3:**
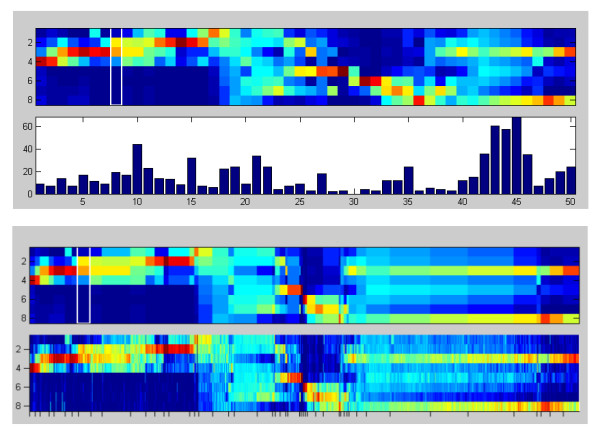
**Prototype plot**. Two alternative display modes of the prototype plot (region 1a and 1b in figure 2): The first mode (top) corresponds to equally spaced prototype profiles with a cluster size bar plot in the lower part. The second mode (bottom) shows the prototypes spaced according to cluster size (upper box) and the normalized intensity profiles of the respective marker candidates (lower box).

The prototype plot shows the array of prototypes according to the current colormap (region 1a) and additional information on the associated marker candidates (region 1b). The vertical axis of region 1a represents the number of data set conditions, while the horizontal axis corresponds to the prototype numbers. A cursor (represented by a white rectangle) marks the current prototype under investigation. By default, the displayed prototype profiles are equally spaced and region 1b shows the associated cluster sizes as a bar diagram. Clicking on the **toggle view** button changes between different graphical representation modes. Besides the default view, the prototype profiles can be spaced according to the size of their associated clusters, which helps to identify dominating intensity profiles (see figure 3). In this case, region 1b contains the original or normalized intensity profiles of the marker candidates associated with each prototype. The title above region 1a indicates, which graphical representation mode is currently activated. By clicking on one of the columns corresponding to a prototype (or using the left and right cursor keys) the associated cluster can be activated.

For the data set of the wounding case study the prototype plot (figure 2, region 1a) reveals

• a block of marker candidates that show high intensities in the conditions representing wt plants only (prototype 1 to 18, condition 1 to 4)

• an intermediate block of different profiles representing high intensities across wt and *dde *2-2 mutant plants (prototype 19 to 24)

• a block of prototypes that show high intensities in the jasmonate deficient mutant plants only (prototype 27 to 36, condition 5 to 8)

• and a block of candidates that particularly represent high concentrations in the third and eighth condition (prototype 40 to 50).

The first block corresponds to clusters in [11] that contain wound induced markers exclusively associated with wild type plants as described in [12,13]. In addition, clusters related to the third block contained markers that seem to be dependent on the jasmonate deficiency.

The corresponding bar diagram (figure 2, region 1b) shows that a number of clusters contain just a few or no marker candidates at all (e.g. cluster 30). These "sparse" clusters result from the restriction of the prototypes on a 1D-topology and usually indicate the use of too many prototypes.

**Figure 4:**
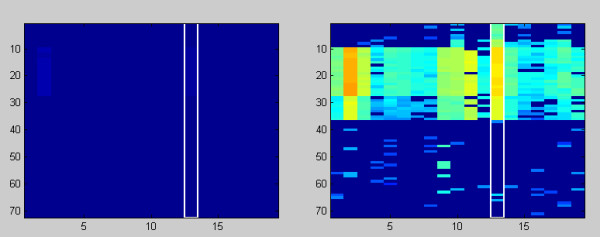
**Cluster plot**. The cluster plot (region 2 in figure 2) with standard (left side) and logarithmical colormapping (right side) of the original intensity values (72 values for each marker candidate according to 8 different conditions and 9 replicas for each condition).

The cluster plot (see figure 2, region 2) shows the intensity profiles of marker candidates in the activated cluster. Each column represents the intensity profile of a single marker candidate displayed according to the current colormap. A cursor (white rectangle) indicates the currently activated candidate. By clicking the **toggle view** button, MarVis switches between normalized and original intensities. By default normalized intensities are shown. The example data set shows large differences regarding the original intensity values. This results in a colormapping, where low original intensities cannot be visually distinguished. When the **Logarithmic intensities** checkbox in the **View** menu is activated, the colormapping for original intensity profiles is calculated logarithmically. This improves the visibility of small intensity differences significantly (see figure 4). The logarithmic transformation is only used for the visualization of original intensity profiles and does not affect the clustering results.

The marker information box (see figure 2, region 3) shows a table of available information on all marker candidates in the currently activated cluster. Each candidate is represented by one row. Apart from marker candidate ID (second column), retention time (rt, third column) and mass-to-charge-ratio (m/z, fourth column), also the values for additional data attributes (see section "Implementation") are displayed. In this example, the column **KWpValue** represents p-values obtained from a Kruskal-Wallis test [24]. These values have been used as a quality measure for selection of marker candidates in [11]. By pressing the up and down cursor keys or selecting a particular row via mouse click, the specific candidate can be highlighted. Within the marker information box, the candidates are sorted by retention time. MarVis keeps this order of the input file when displaying the candidates of single clusters.

The marker scatter plot (see figure 2, region 4) displays the rt vs. m/z values of each marker candidate in the currently activated cluster using big red dots. The currently activated candidate is represented by a big blue dot. In the background all marker candidates of the data set are plotted as small gray dots. By clicking into the plot a particular candidate can be activated. Using the scatter plot, putative isotopomers or adducts of compounds can easily be identified by vertical stacks of candidates that do not differ in retention time (see figure 2, region 4).

The active-prototype/marker plot (see figure 2, region 5) by default displays the magnified prototype of the activated cluster according to the current colormap.

### Additional functionality

MarVis stores a list of selected marker candidates in memory. Single candidates or entire clusters can easily be added to or removed from this list. The selected marker candidates can be exported as a CSV file for further analysis, e.g. for identification of related metabolic pathways based on mass values of candidates [25]. The candidates may also be re-clustered using a lower number of prototypes in order to avoid sparse clusters. In addition to the export of selected marker candidates, MarVis can save the entire clustering result as a CSV file. This includes the marker data with additional values for normalized marker candidates (sorted by cluster order), cluster number, and intensity profiles of the associated prototypes. The current settings, which include the data set and all user-specific parameters (e.g. current colormap, visualization properties, dialog entries), can be saved and restored. For details on the above-mentioned functions see the MarVis documentation.

## Conclusion

MarVis provides a graphical user interface for exploratory data analysis, well-suited for the visualization of metabolomic intensity profiles. The realization of 1D-SOMs gives a convenient overview of multivariate data sets. In particular, the specialized visualization effectively supports researchers to cope with the problem of an unknown number of biologically meaningful groups of intensity profiles. In that way, interesting groups can easily be identified based on their intensity patterns and their position in the prototype array. Additional data attributes that support the analysis and interpretation of marker candidates can be integrated in MarVis using customized data fields in the CSV input file. By using the CSV export functions, the clustering results can be imported and processed by other statistical analysis software. The customizable CSV file format also allows to import, cluster and analyze experimental data from other than metabolomic studies, e.g. from gene expression experiments. An example application on gene expression data is shown in the MarVis documentation.

## Availability and requirements

• **Project name**: MarVis

• **Project home page**: 

• **Operating system(s)**: Microsoft^® ^Windows XP/Vista and Linux x86

• **Programming language**: Matlab^®^

• **Other requirements**: Matlab^® ^Compiler Runtime 7.8 (provided with MarVis)

• **License**: Free for academic use

## Authors' contributions

AK implemented the MarVis graphical user interface and drafted parts of the manuscript. TL contributed conceptually and drafted parts of the manuscript. KF, CG and IF provided the metabolomic case study data set, tested the software, contributed conceptually and drafted parts of the manuscript. PM implemented the clustering algorithm and drafted parts of the manuscript. All authors read and approved the final manuscript.
